# Circulating estradiol and its biologically active metabolites in endometriosis and in relation to pain symptoms

**DOI:** 10.3389/fendo.2022.1034614

**Published:** 2023-01-18

**Authors:** Jean-Philippe Emond, Patrick Caron, Maja Pušić, Véronique Turcotte, David Simonyan, Andrej Vogler, Joško Osredkar, Tea Lanišnik Rižner, Chantal Guillemette

**Affiliations:** ^1^ Pharmacogenomics Laboratory, Centre Hospitalier Universitaire (CHU) de Québec – Université Laval Research Center and Faculty of Pharmacy, Université Laval, Québec City, QC, Canada; ^2^ Laboratory for Molecular Basis and Biomarkers of Hormone Dependent Diseases, Institute of Biochemistry, Medical Faculty, University of Ljubljana, Ljubljana, Slovenia; ^3^ Statistical and Clinical Research Platform, CHU de Québec – Université Laval Research Center, Québec City, QC, Canada; ^4^ Department of Obstetrics & Gynaecology, University Medical Centre Ljubljana, Ljubljana, Slovenia; ^5^ Clinical Institute of Clinical Chemistry and Biochemistry, University Medical Centre Ljubljana, Ljubljana, Slovenia; ^6^ Canada Research Chair in Pharmacogenomics, Université Laval, Québec City, QC, Canada

**Keywords:** endometriosis, catechol estrogens, steroids, mass spectrometry, pain symptoms

## Abstract

**Objectives:**

Endometriosis (EM) is an estrogen-dominant inflammatory disease linked to infertility that affects women of reproductive age. EM lesions respond to hormonal signals that regulate uterine tissue growth and trigger inflammation and pain. The objective of this study was to evaluate whether estradiol (E_2_) and its biologically active metabolites are differentially associated with EM given their estrogenic and non-estrogenic actions including proliferative and inflammatory properties.

**Design:**

We performed a retrospective study of 209 EM cases and 115 women without EM.

**Methods:**

Pain-related outcomes were assessed using surveys with validated scales. Preoperative serum levels of estradiol (E_2_) and estrone (E_1_), their 2-, 4- and 16- hydroxylated (OH) and methylated (MeO) derivatives (n=16) were measured by mass spectrometry. We evaluated the associations between estrogen levels and EM anatomic sites, surgical stage, risk of EM, and symptoms reported by women. Spearman correlations established the relationships between circulating steroids.

**Results:**

Of the sixteen estrogens profiled, eleven were detected above quantification limits in most individuals. Steroids were positively correlated, except 2-hydroxy 3MeO-E_1_ (2OH-3MeO-E_1_). Higher 2OH-3MeO-E_1_ was linked to an increased risk of EM (Odd ratio (OR)=1.91 (95%CI 1.09-3.34); *P*=0.025). Ovarian EM cases displayed enhanced 2-hydroxylation with higher 2MeO-E_1_ and 2OH-E_1_ levels (*P*< 0.009). Abdominal, pelvic and back pain symptoms were also linked to higher 2OH-3MeO-E_1_ levels (OR=1.86; 95%CI 1.06-3.27; *P*=0.032).

**Conclusions:**

The 2-hydroxylation pathway emerges as an unfavorable feature of EM, and is associated with ovarian EM and pain related outcomes.

## Introduction

Endometriosis (EM) affects approximately 10% of women of reproductive age (1) and is defined by the presence of endometrial glands or stoma outside the uterine cavity. It is a non-malignant disease nonetheless associated with dysmenorrhea, dyspareunia, pelvic pain and infertility due to the presence of ectopic tissue and inflammation (2–4). Endometriotic lesions can be superficial peritoneal, ovarian or deeply infiltrating (5, 6). The etiology of endometriosis is complex (7) and multifactorial thus several theories have been proposed to explain the clinical manifestation of endometriosis; Sampson’s theory of retrograde menstruation, coelomic metaplasia theory, Mullerian rests theory, stem cell theory, impaired immune system theory, and others (8). Endometriosis is considered a chronic inflammatory disease with altered peritoneal environment in patients with endometriosis. The ectopic lesions recruit immune cells which leads to production of pro-inflammatory molecules and cytokines and also promote angiogenesis and innervation and thus contribute to survival of these lesions (9).

EM is an estrogen-dependent disease with molecular hallmarks of genetic predisposition, altered hormonal milieu (estrogen dependence and progesterone resistance) and inflammation (10, 11). Changes in steroid biotransformation pathways have been reported leading to an increased local production of estrogens in endometriosis lesions (12). EM lesions respond to hormonal signals such as estradiol (E_2_) that regulate uterine tissue growth and triggers inflammation, and that are linked to pain symptoms (13). Excessive inflammation also leads to changes in sex steroid receptors (ERα and ERβ) expression and enhanced estrogen biosynthesis in endometriotic lesions, involving aromatase, sulfatase and other pathways (14–21). Treatment of pelvic pain in EM thus includes the use of nonsteroidal anti-inflammatory drugs, oral contraceptives and progestins.

Estrogens comprise a vast array of hydroxylated (OH) and methoxylated (MeO) catechol estrogen (CE) metabolites with diverse biological activities. The synthesis of CE metabolites from E_2_ and estrone (E_1_) involves various metabolic routes, namely the 2-hydroxylation (2OH), 4-hydroxylation (4OH) and 16-hydroxylation (16OH) pathways and the action of the catechol-O-methyltransferase (COMT) to form 2- and 4- MeOCEs (22, 23). Besides acting as ligands of ERα and ERβ, CEs also present non-estrogenic properties (22, 24). MeOCEs have antiangiogenic and antiproliferative actions whereas 4-OHCEs have procarcinogenic properties (22, 25–27). Both the 2OH and 4OH CE derivatives generally have reduced estrogenic effects (24, 28) as opposed to the 16OH pathway that retains most of its estrogenic properties, with a preferential action on the ERβ (24). In addition, E_2_ was previously found to be associated with pain due to its effects on nerves and inflammation (13). Some CEs present estrogenic activities resembling E_2_ and they may be prone to cause pain.

Given the suspected biological roles of catechol estrogens, and their associations with several hormone-sensitive diseases including endometrial cancer (29, 30), we hypothesized that circulating levels of E_2_ and/or its biologically active metabolites were associated with an altered risk of EM (primary objective) and severity of pain symptoms (secondary objective).

## Material and methods

### Study cohort

**Figure 1:**
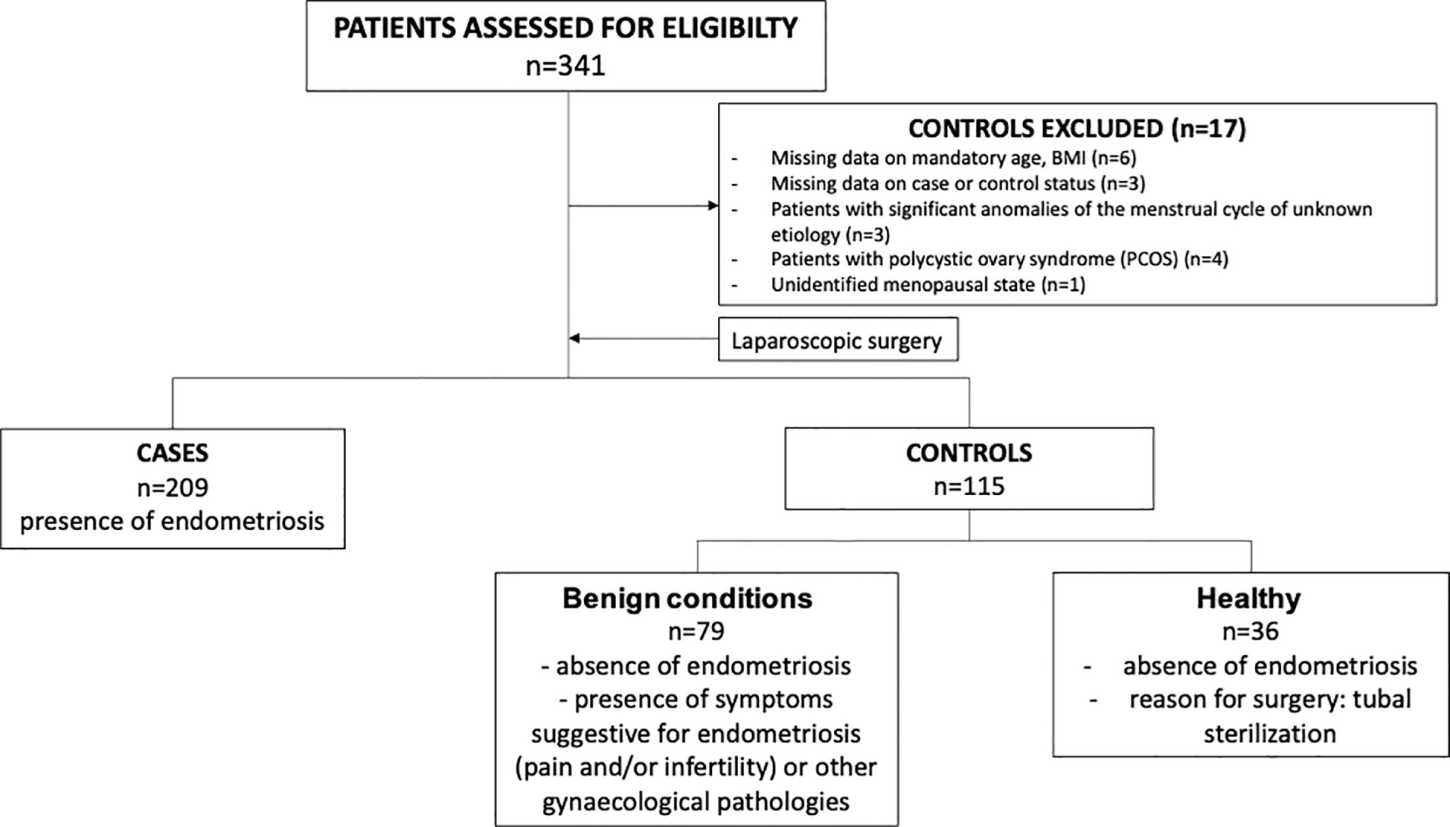
Flowchart of patient cohort.

The study design corresponded to a retrospective case-control study comprising cases and controls from the same type of population (31). Part of this cohort was described previously (32–34). Patients’ enrolment took place from March 2008 to June 2018 at the Departments of Obstetrics and Gynecology at the University Medical Centre Ljubljana, Slovenia. The study comprised patients who visited gynecologist with problems/symptoms that are indicative for laparoscopy surgery. The inclusion criteria were an indication for a diagnostic laparoscopy for symptoms suggestive of EM such as pain, infertility, ovarian cysts, other gynecological pathologies such as myomas and tubal sterilization. The exclusion criteria were pregnancy, age below 18 years, menopausal status, gynecological malignancies, cancelled surgery, previous hysterectomy, drug abuse and HIV infection (32). Of the 341 women, 17 participants were excluded to manage confounders: six because of missing data on mandatory age and/or BMI, three due to unknown case or control status, four with polycystic ovary syndrome (PCOS) that could impact hormone levels, one due to a prior unidentified menopausal state, and three with significant anomalies of the menstrual cycle of unknown etiology. The remaining cohort of 324 women underwent either diagnostic laparoscopy or laparoscopic tubal sterilization and were divided according to presence (n=209, cases) or absence (n=115, controls) of EM. Controls were further divided into two groups (patients with benign pathologies (n=79) and healthy controls (n=35). Patients with benign pathologies had symptoms suggestive for EM (infertility and/or pain) or other gynecological pathologies. Healthy patients underwent laparoscopic tubal sterilization and had no symptoms suggestive for EM (**Figure 1**). A post-hoc ANOVA power analysis test (power package and R Statistical Software v4.1.2; R Core Team 2021) estimated that a sample size per group of 36 was suffcient). The clinical characteristics presented in **Table 1** included age, body mass index (BMI), type of EM (ovarian, ovarian and peritoneal, peritoneal, and deep infiltrating), rASRM stage of disease (35), smoking status (current, former or never), use of hormonal therapy (last three months), use of oral contraception (last three months), and endometrial phase (secretory or proliferative). Patient-filled surveys using validated numeric rating scales documented the outcomes of “abdominal, pelvic and back pain”, “dysmenorrhea (frequency)”, “dysmenorrhea (intensity)”, “score of dysmenorrhea”, “dyspareunia (frequency)”, “dyspareunia (intensity)” and “dysuria or dyschezia (frequency)” (36, 37). For EM cases, data for pain-related outcomes were available for 98.6% to 99.5% of participants, except for the “score of dysmenorrhea’’ (61.2%), “dysmenorrhea (intensity)” (35.4%), and “dyspareunia (intensity)” (59.3%) outcomes. In control cases, data was available for 86.7% to 97.4% of participants, except for “score of dysmenorrhea’’ (56.5%) and “dyspareunia (intensity)” (55.7%) outcomes. The patient’s characteristics related to pain symptoms are presented in **Supplementary Table 1**. Pain related outcomes were dichotomized for the statistical analysis. The dichotomization for “abdominal, pelvic and back pain” was “yes or no”. For “dysmenorrhea (frequency)”, “dyspareunia (frequency)” and “dysuria or dyschezia (frequency)”, the dichotomization was “infrequent (never, almost never or sometimes) or frequent (quite often or very often)”. For “dyspareunia (intensity)” and “dysmenorrhea (intensity)”, the dichotomization was “mild (no or slight pain) or moderate to severe (medium or strong pain)”. For the “score of dysmenorrhea”, the dichotomization was “scores of ≤5 or of >5”. All participants provided an informed consent prior to their enrolment. This study was conducted in accordance with the declaration of Helsinki. This study was approved by the National Medical Ethics Committee in Slovenia (#0120-127/2016/6) and the ethics committee of the CHUQc – Université Laval (#2012-993).

**Table 1:** Characteristics and clinical data of endometriosis cases (n=209) and controls (n=115).

	Cases (n=209)	Controls (n=115)		
		All	Benign pathologies (n=79)	Healthy (n=36)
Age^1^				
Mean age ± SD (years)	31.57 ± 5.35	34.66 ± 6.79	32.03 ± 6.42	40.44 ± 2.87
Body mass index (BMI)^1^				
Mean ± SD	22.42 ± 3.66	24.88 ± 4.55	24.53 ± 4.67	25.66 ± 4.26
Type of disease				
Ovarian	35 (16.7%)	–	–	–
Ovarian and peritoneal	59 (28.2%)	–	–	–
Peritoneal	62 (29.7%)	–	–	–
Deep infiltrating	53 (25.4%)	–	–	–
Stage of disease				
1	49 (23.4%)	–	–	–
2	38 (18.2%)	–	–	–
3	67 (32.1%)	–	–	–
4	40 (19.1%)	–	–	–
Missing	15 (7.2%)	–	–	–
Smoking status				
Current	68 (32.5%)	37 (32.2%)	27 (34.2%)	10 (27.8%)
Former	11 (5.3%)	14 (12.2%)	9 (11.4%)	5 (13.9%)
Never	129 (61.7%)	62 (53.9%)	42 (53.2%)	20 (55.6%)
Missing	1 (0.5%)	2 (1.7%)	1 (1.3%)	1 (2.8%)
Hormonal therapy^2^				
Yes	38 (18.2%)	17 (14.8%)	15 (19.0%)	2 (5.6%)
No	171 (81.8%)	93 (80.9%)	62 (78.4%)	31 (86.1%)
Missing		5 (4.3%)	2 (2.5%)	3 (8.3%)
Per oral contraception^1,2^				
Yes	41 (19.6%)	27 (23.5%)	12 (15.2%)	15 (41.7%)
No	168 (80.4%)	85 (73.9%)	65 (82.3%)	20 (55.6%)
Missing	0 (0.0%)	3 (2.6%)	2 (2.5%)	1 (1.5%)
Endometrial phase^1^				
Secretory	83 (39.7%)	48 (41.7%)	28 (35.4%)	20 (55.6%)
Proliferative	112 (53.6%)	53 (46.1%)	38 (48.1%)	15 (41.7%)
Oral hormonal contraceptive	14 (6.7%)	11 (9.6%)	11 (13.9%)	0 (0.0%)
Missing	0 (0.0%)	3 (2.6%)	2 (2.5%)	1 (2.8%)

Surgical stages were classified using the revised American Society for Reproductive Medicine score (rASRM) classification of endometriosis. ^1^ *P*-values <0.0001 obtained using Pearson’s chi square or Fisher’s exact test when appropriate. ^2^Last 3 months.

### Quantification of estrogen derivatives

Blood samples were collected two days prior to surgery as described (32), and following strict standard operating procedures for collection, processing and storage at -80˚C to preserve stability of metabolites such as steroids. Briefly, 4 ml of blood sample was collected by venipuncture from the median cubital vein using BD Vacutainer tubes (#369032; Becton Dickinson and Company, NJ, USA). The collected samples were incubated for no more than 1 h at room temperature and then centrifuged at 1400 × g for 10 min at room temperature. The seperated serum was collected, aliquoted, and stored at -80°C until analysis. Only samples that were frozen/thawed once were used for analysis. A specific set of 16 estrogen derivatives were quantified in 250 μL of serum using a liquid-chromatography tandem mass spectrometry assay (LC-MS/MS) as described (29). The lower limit of quantification (LLOQ) was 5 pg/mL. Sums including all analytes and metabolic ratios were calculated for the different metabolic pathways. Catechol estrogens at levels below LLOQ (even if detected above the limit of detection) were considered undetected.

### Statistical analysis

Differences in estrogen hormone levels between cases and controls, anatomic sites and surgical stages were determined by bivariate analyses on means of log transformed continuous hormone levels. The relationship between hormone levels was assessed using Spearman’s rank-order correlation. Odd ratios (OR) were obtained using dichotomized hormone levels (independent variables) based on the median levels of controls as performed in previous studies (29) in a multivariate logistic regression model, adjusted for age, BMI, smoking status, oral contraception (last three months), hormonal therapy (last three months) and the endometrial phase (secretory or proliferative). Logistic models and Fisher’s scoring were used to determine the ORs for pain related outcomes in cases. *P*-values were obtained using Pearson’s chi square, Fisher’s exact test, the one-way analysis of covariance F-test corrected with Tukey when appropriate or the Spearman Rho statistic test in the appropriate contexts. Results were considered statistically significant when *P* < 0.05. Statistical analyses were performed by the statistician (DS) using the software SAS 9.4 by SAS Institute Inc. (Cary, NC, USA). Due to the exploratory nature of the study, no adjustment for multiple comparison was done.

## Results

Characteristics of EM and controls are despicted in **Table 1**. A total of 16 estrogen derivatives were quantified by MS in the serums of 341 women. Most estrogens and their oxidative metabolites (11 out of 16) were above LLOQ, except for 4OH-E_2_, 17epi-E_3_, 2MeO-E_2_, 4MeO-E_1_ and 4MeO-E_2_ detected in less than 12% of the cohort. These five estrogens were thus excluded in subsequent statistical analyses (**Supplementary Table 2**). Levels of estrogens are displayed in **Table 2** with E_1_, E_2_, 2OH-E_1_, E_3_, 16αOH-E_1_ and 16keto-E_2_ displaying the highest levels. In addition, 2OH-3MeOE_1_ levels were higher in cases compared to controls (by 123%; *P* = 0.02) (**Table 2**). The analysis of hormone levels according to anatomic sites of EM (**Table 3**), showed higher levels of 2OH-E_1_ (by 18%; *P* = 0.009), 2MeO-E_1_ (38%; *P* = 0.002), sum of 2OH (7%; *P* = 0.013), sum of MeO (23%; *P* = 0.036), ratio of 2OH/sum E_1_/E_2_ (24%; *P* = 0.023), and ratio of 2OH-E_1/_16αOH-E_1_ (101%; *P* = 0.033) in cases diagnosed with ovarian EM. No evidence of an association was observed in relation to surgical stage (**Supplementary Table 3**).

**Table 2:** Steroid levels for EM cases (n=209) and for controls (n=115) that included subjects with benign pathologies and healthy controls.

	Cases (n=209)	Controls (n=115)						
			All		Benign pathologies (n=79)		Healthy (n=36)	
Steroids	Median	10-90%	Median	10-90%	Median	10-90%	Median	10-90%
E_1_	831.00	203.00-2830.00	882.00	262.00-2730.00	788.00	149.00-2830.00	1004.50	325.00-2130.00
E_2_	138.00	8.86-396.00	118.00	17.20-320.00	100.00	13.20-320.00	163.50	87.70-345.00
2OH-E_1_	96.40	9.70-247.00	93.10	11.70-227.00	73.90	11.40-212.00	123.50	12.60-262.00
2OH-E_2_	10.60	2.50-37.90	12.40	2.50-41.10	10.40	2.50-27.90	17.55	2.50-48.80
4OH-E_1_	8.77	2.50-25.90	7.59	2.50-22.50	7.65	2.50-25.00	7.42	2.50-21.50
16αOH-E_1_	32.10	7.36-140.00	44.60	7.91-142.00	38.30	6.06-142.00	61.60	15.30-261.00
16epi-E_3_	8.07	2.50-29.00	10.30	2.50-28.80	8.31	2.50-23.70	13.30	5.50-33.40
**16keto-E_2_**	**31.70**	**8.05-130.00**	**40.80**	**7.07-142.00**	**31.10**	**6.55-92.00**	58.00*	**22.50-269.00**
E_3_	67.00	14.90-256.00	85.80	14.70-265.00	68.10	11.90-236.00	121.50	42.80-311.00
2MeO-E_1_	24.50	2.50-80.90	18.40	2.50-60.60	15.30	2.50-61.60	25.35	2.50-60.60
**2OH-3MeO-E_1_**	5.58*	**2.50-16.00**	**2.50**	**2.50-13.60**	**2.50**	**2.50-12.10**	**2.50**	**2.50-20.60**
Sums								
E_1_/E_2_	1009.00	219.45-3102.00	1019.00	296.30-3040.00	885.40	182.33-3166.00	1138.50	460.00-2457.00
2OH	104.50	12.20-276.00	99.90	14.20-260.80	83.80	13.90-238.90	146.45	15.10-291.70
4OH	11.30	5.00-30.60	10.20	5.00-30.20	10.15	5.00-30.50	11.18	5.00-30.20
**16OH**	**152.10**	**33.70-551.30**	**204.11**	**37.36-591.80**	**140.21**	**31.88-428.20**	264.90*	**69.24-758.90**
MeOs	41.19	16.33-99.39	33.00	15.83-79.51	30.00	15.83-87.60	39.07	15.63-79.30
OHs	302.20	54.62-837.80	358.63	69.23-801.10	288.25	57.52-648.80	474.50	211.26-941.60
CEs	352.40	81.72-928.61	390.22	98.36-876.50	312.61	82.52-734.78	539.77	242.16-1015.68
Ratios								
2OH/sum E_1/_E_2_	0.09	0.04-0.22	0.09	0.03-0.23	0.08	0.03-0.20	0.10	0.03-0.33
4OH/sum E_1/_E_2_	0.01	0.00-0.03	0.01	0.00-0.03	0.01	0.00-0.04	0.01	0.00-0.02
16OH/sum E_1/_E_2_	0.15	0.06-0.45	0.15	0.07-0.52	0.13	0.06-0.41	0.21	0.11-0.60
CEs/sum E_1/_E_2_	0.35	0.18-0.72	0.34	0.16-0.84	0.29	0.15-0.68	0.41	0.21-0.96
**2OH/4OH**	**8.14**	**2.06-20.2**	**9.09**	**2.13-22.8**	**8.38**	**1.74-22.32**	11.46*	**3.02-29.70**
2OH/16OH	0.68	0.14-1.55	0.63	0.12-1.36	0.66	0.12-1.42	0.53	0.09-1.36
2OH/MeOs	2.46	0.80-4.08	2.79	0.71-5.00	2.71	0.71-4.84	3.13	1.21-5.35
4OH/16OH	0.08	0.02-0.27	0.06	0.01-0.24	0.08	0.02-0.34	0.05	0.01-0.13
4OH/MeOs	0.27	0.12-0.60	0.29	0.14-0.65	0.29	0.15-0.78	0.30	0.13-0.44
**OHs/MeOs**	**6.43**	**2.51-16.8**	**8.06**	**3.07-18.61**	**7.46**	**2.48-16.91**	10.91*	**4.57-27.51**
2OH/16αOH-E_1_	2.61	0.38-8.15	2.42	0.43-6.00	2.46	0.44-6.00	2.24	0.35-7.22

Steroids are reported in pg/mL. The sum of hydroxy derivatives (OHs) includes 2OH, 4OH and 16OH. The sum of all catechol estrogens (CEs) includes OHs and MeOs. *P*-values were obtained based on the one-way analysis of covariance F test with correction when necessary. Continuous means of hormone levels were log transformed and adjusted for age and BMI for statistical analysis. Significant *P* values <0.05 are highlighted with a star (*) and text in bold. The median values and the 10-90% intervals for E_2_ were 123.00 (31.6 -417.0 pg/mL) for women in the proliferative phase and 169.00 (13.20-333.00 pg/mL) for women in the secretory phase.

**Table 3:** Steroid levels according to anatomic sites of disease in 209 endometriosis cases.

Steroids (pg/mL)	Median (10-90%)				*P-*value			
	Ovarian	Ovarian peritoneal	Peritoneal	Deep infiltrating	All	1 vs 3	1 vs 2	1 vs all
	n=35	n=59	n=62	n=53				
E_1_	908.00 (258.00-3420.00)	723.00 (188.00-2990.00)	777.00 (188.00-3190.00)	873.00 (306.00-2160.00)				
E_2_	143.00 (30.20-649.00)	138.00 (6.61-393.00)	130.50 (9.17-438.00)	139.00 (14.40-239.00)				
2OH-E_1_	111.00 (53.70-455.00)	87.40 (7.06-211.00)	98.20 (7.76-234.00)	96.40 (20.40-196.00)	**0.033**	**0.022**	**0.011**	**0.009**
2OH-E_2_	12.50 (2.50-67.40)	8.62 (2.50-36.60)	11.65 (2.50-33.50)	9.58 (2.50-35.20)				0.088
4OH-E_1_	9.67 (2.50-35.10)	8.78 (2.50-24.40)	7.94 (2.50-22.10)	8.71 (2.50-24.20)				0.086
16αOH-E_1_	28.70 (7.36-197.00)	27.20 (7.04-179.00)	39.00 (8.22-139.00)	34.70 (7.54-120.00)				
16epi-E_3_	8.51 (2.50-29.20)	7.55 (2.50-31.00)	8.39 (2.50-28.10)	7.84 (2.50-25.20)				
16keto-E_2_	40.50 (11.90-137.00)	34.10 (6.32-163.00)	26.80 (7.85-94.70)	30.00 (8.02-119.00)				
E_3_	79.00 (23.70-233.00)	59.20 (14.90-245.00)	69.60 (9.06-329.00)	62.8 (17.00-225.00)				
2MeO-E_1_	29.70 (10.80-234.00)	20.10 (2.50-75.50)	20.90 (2.50-63.20)	23.90 (5.48-80.90)	**0.011**	0.076	**0.029**	**0.002**
2OH-3MeO-E_1_	5.65 (2.50-16.30)	2.50 (2.50-16.00)	2.50 (2.50-15.40)	6.05 (2.50-15.40)				
Sums								
E_1_/E_2_	1057.00 (295.20-3772.00)	857.00 (192.61-3474.00)	903.50 (196.86-3490.00)	1024.00 (351.80-2389.00)				0.058
2OH	117.70 (56.20-573.20)	98.77 (9.56-254.70)	126.55 (10.26-254.60)	104.50 (22.90-225.20)	0.083			**0.013**
4OH	13.80 (5.00-41.70)	11.37 (5.00-29.20)	10.44 (5.00-25.10)	11.21 (5.00-28.40)				
16OH	172.02 (45.00-540.10)	152.10 (33.70-682.20)	148.86 (32.51-542.00)	146.96 (32.26-444.90)				
MeOs	48.00 (26.20-269.56)	36.59 (16.33-99.39)	39.20 (12.50-80.10)	41.20 (15.48-103.70)				**0.036**
OHs	362.63 (114.39-1212.27)	267.50 (46.66-837.80)	299.54 (49.93-820.30)	343.91 (70.83-689.70)				
All CEs	404.57 (130.00-1283.07)	306.10 (79.50-885.49)	342.84 (72.00-850.37)	386.94 (102.43-738.61)				0.082
Ratios								
2OH/sum E_1/_E_2_	0.11 (0.04-0.27)	0.09 (0.03-0.18)	0.08 (0.03-0.20)	0.10 (0.04-0.22)	0.078			**0.023**
4OH/sum E_1/_E_2_	0.01 (0.00-0.03)	0.01 (0.00-0.04)	0.01 (0.00-0.03)	0.01 (0.01-0.03)				
16OH/sum E_1/_E_2_	0.15 (0.07-0.30)	0.15 (0.06-0.38)	0.17 (0.08-0.48)	0.13 (0.06-0.58)				
CEs/sum E_1/_E_2_	0.32 (0.19-0.63)	0.36 (0.14-0.72)	0.34 (0.21-0.70)	0.34 (0.17-0.83)				
2OH/4OH	10.37 (2.87-23.33)	7.36 (1.00-17.20)	8.46 (1.77-26.80)	8.14 (3.02-18.93)				
2OH/16OH	0.89 (0.28-1.43)	0.58 (0.13-1.44)	0.55 (0.13-1.55)	0.76 (0.13-1.91)				0.090
2OH/MeOs	2.54 (1.22-4.50)	2.38 (0.49-3.90)	2.67 (0.49-4.46)	2.23 (0.88-3.90)				
4OH/16OH	0.09 (0.03-0.24)	0.09 (0.02-0.27)	0.07 (0.02-0.23)	0.09 (0.01-0.34)				
4OH/MeOs	0.24 (0.11-0.73)	0.28 (0.14-0.68)	0.27 (0.16-0.59)	0.29 (0.12-0.55)				
OHs/MeOs	6.32 (2.96-10.22)	5.96 (2.02-18.54)	7.42 (2.27-15.43)	5.79 (2.70-18.54)				0.075
2OH-E_1_/16αOH-E_1_	4.37 (1.29-8.69)	2.13 (0.22-7.64)	1.86 (0.35-5.88)	2.61 (0.51-8.79)				**0.033**

The sum of hydroxy derivatives (OHs) includes 2OH, 4OH and 16OH. The sum of all catechol estrogens (CEs) includes OHs and MeOs. Data represent bivariate analysis corrected with Tukey and adjusted for age and BMI. *P*-values were obtained with the F test using log transformed data. Only trends (*P*<0.1) and significant associations (*P*<0.05; in bold) are displayed.

The first objective aimed to establish the potential association of estrogens with the risk of developing EM. Women with higher circulating levels of specific catechol estrogens were shown to be more predisposed to EM risk in multivariable analysis adjusted for confounders including age, BMI, tobacco status, contraception, hormonal therapy and endometrial phase, which differ in EM cases compared to controls (**Table 1**). More specifically, women with higher levels of 2OH-3MeO-E_1_ had an adjusted OR of 1.91 (95%CI 1.09-3.34; *P* =0.025). This finding was also observed when restricted to healthy subjects and controls with benign pathologies (OR =2.61 (95%CI 0.84–8.09); *P* =0.097 and 1.56 (95%CI 0.84–2.91); *P* =0.164) but did not reached significance. A lower risk of EM was observed in association with elevated 16OH derivatives (with OR values of 0.22 for 16epi-E_3_ (95%CI 0.06–0.83); *P* =0.025) and 0.22 for 16keto-E_2_ (95%CI 0.06–0.84); *P* =0.027). Since 2OH-3MeO-E_1_ was the main metabolite associated with the risk of EM, we evaluated whether the correlation of this metabolite with the other estrogens was different between controls and cases. We observed that 2OH-3MeO-E_1_ was weakly but significantly correlated with 2-OH derivatives in EM cases at 0.20 (*P*<0.05) but not in controls (**Table 4**).

**Table 4:** Spearman correlation coefficients among endogenous hormone levels in endometriosis cases (EM), benign conditions (BC) and healthy (H).

		E_1_	E_2_	2OH-E_1_	2OH-E_2_	4OH-E_1_	16αOH-E_1_	16epi-E_3_	16keto-E_2_	E_3_	2MeO-E_1_	3MeO-E_1_
E_1_	EM		0.81*	0.71*	0.60*	0.57*	0.67*	0.72*	0.72*	0.69*	0.71*	0.09
	BC		0.77*	0.67*	0.49*	0.46*	0.65*	0.69*	0.50*	0.58*	0.70*	0.00
	H		0.68*	0.53*	0.33*	0.68*	0.41*	0.64*	0.31	0.37*	0.59*	0.21
E_2_	EM			0.73*	0.62*	0.56*	0.66*	0.71*	0.74*	0.72*	0.70*	0.07
	BC			0.71*	0.58*	0.47*	0.75*	0.74*	0.69*	0.67*	0.74*	-0.02
	H			0.42*	0.26	0.60*	0.55*	0.53*	0.50*	0.31	0.57*	-0.09
2OH-E_1_	EM				0.82*	0.57*	0.52*	0.57*	0.56*	0.53*	0.86*	0.20*
	BC				0.89*	0.53*	0.49*	0.57*	0.38*	0.45*	0.88*	-0.01
	H				0.87*	0.57*	0.22	0.41*	0.04	0.17	0.80*	-0.08
2OH-E_2_	EM					0.47*	0.48*	0.62*	0.46*	0.56*	0.63*	0.15*
	BC					0.45*	0.36*	0.52*	0.34*	0.43*	0.72*	0.01
	H					0.50*	-0.03	0.27	-0.03	0.15	0.64*	0.17
4OH-E_1_	EM						0.31*	0.36*	0.41*	0.35*	0.58*	0.08
	BC						0.22*	0.25*	0.17	0.17	0.58*	-0.06
	H						0.20	0.28	0.16	0.03	0.67*	0.05
16αOH-E_1_	EM							0.80*	0.78*	0.82*	0.49*	0.01
	BC							0.85*	0.81*	0.82*	0.49*	-0.11
	H							0.69*	0.73*	0.60*	0.28	-0.38*
16epi-E_3_	EM								0.81*	0.92*	0.47*	0.09
	BC								0.79*	0.92*	0.52*	-0.08
	H								0.56*	0.84*	0.34*	-0.26
16keto-E_2_	EM									0.85*	0.51*	0.14*
	BC									0.79*	0.34*	-0.01
	H									0.51*	0.14	-0.10
E_3_	EM										0.45*	0.09
	BC										0.38*	-0.06
	H										0.09	-0.18
2MeO-E_1_	EM											0.14*
	BC											0.02
	H											-0.09
2OH-3MeO-E_1_	EM											
	BC											
	H											

Correlation values were similar with all controls (when BC and H were analyzed together). The *P*-values of <0.05 are identified with a (*) and highlighted in bold. Correlations were tested using the Spearman Rho statistic test.

A secondary objective aimed to explore the relationship between hormone levels and symptoms of pain in EM cases (**Table 5**). Higher levels of 2OH-3MeO-E_1_ were associated with the risk of pain in the abdominal, pelvic and back regions (OR =1.86 (95%CI 1.06-3.27) *P* =0.032). Higher levels of 16αOH-E_1_ were inversely associated with the risk of pain in the abdominal, pelvic and back regions (OR =0.55 (95%CI 0.31-0.97); *P* =0.038). More frequent menstrual pain was associated with elevated E_1_/E_2_ (OR = 1.90 (95%CI 1.03-3.51); *P* =0.041) whereas more severe menstrual pain was linked to a higher metabolic ratio of 4OH/sum of E_1_/E_2_ (OR =1.95; (95%CI 1.05-3.61); *P* =0.035) (**Table 5**). Higher levels of 16keto-E_2_ were associated with the risk of more severe dyspareunia experienced in the last three months (OR = 2.40 (95%CI 1.02-5.62); *P* =0.045. Higher levels of 4OH-E_1_ were associated with a reduced risk dysuria or dyschezia experienced in the last three months (OR =0.32 (95%CI 0.12-0.89); *P* =0.028) (**Supplementary Table 4**).

**Table 5:** Significant associations between pain and steroid levels in EM cases (n=209).

Steroids	Comparator Medians in pg/mL (n)	Outcome Medians in pg/mL (n)	OR (95%CI)	*P*-value
Abdominal, pelvic, and back pain (n=207)				
16αOH-E_1_	No 42.60 (115)	Yes 26.15 (92)	0.55 (0.31-0.97)	**0.038**
2OH-3MeO-E_1_	No 2.50 (115)	Yes 5.99 (92)	1.86 (1.06-3.27)	**0.032**
Dysmenorrhea (frequency) (n=208)				
sum E_1/_E_2_	Infrequent 857.00 (67)	Frequent 1064.90 (141)	1.90 (1.03-3.51)	**0.041**
Dysmenorrhea (intensity) (n=74)				
Ratio 4OH/sum E_1/_E_2_	Mild 0.01 (45)	Moderate to severe 0.01 (29)	1.95 (1.05-3.61)	**0.035**
Dyspareunia (intensity) (n=124)				
16keto-E_2_	Mild 26.90 (95)	Moderate to severe 46.60 (29)	2.40 (1.02-5.62)	**0.045**
Dysuria or dyschezia (frequency) (n=208)				
4OH-E_1_	Infrequent 8.87 (189)	Frequent 2.50 (19)	0.32 (0.12-0.89)	**0.028**

The sum of hydroxy derivatives (OHs) includes 2OH, 4OH and 16OH. The sum of all catechol estrogens (CEs) includes OHs and MeOs. Odds ratios (OR) and their P-values were obtained using a logistic regression model adjusted for age and BMI. The number of cases with data on clinical outcomes are identified next to each outcome. There was no evidence of an association for the “score of dysmenorrhea” and the “dyspareunia (frequency)” outcomes. Significant P-values (<0.05) are shown in bold.

## Discussion

EM is a complex estrogen-sensitive condition characterized by a chronic inflammation process for which the potential role of estrogen metabolites remains to be fully investigated. We report that higher levels of 2OH-3MeO-E_1_ were associated with an increased risk of EM, with an approximately two-fold higher median level observed in circulation of EM cases compared to controls. An enrichment of the 2OH metabolic pathway, with significantly higher levels of 2OH-E_1_, 2MeO-E_1_, sum of MeO and ratio of 2OH-E_1/_16αOH-E_1_, was also observed in ovarian EM cases compared to cases affected with lesions at other anatomical sites. A perturbation of estrogen metabolism (2OH-3MeO-E_1_ and 16αOH-E_1_) was further associated with pain symptoms.

Estrogens and their receptors play a key role in the pathophysiology of EM. Studies reported higher levels of systemic and locally synthesized estrogens in EM cases promoting the growth of lesions (38). This increase in estrogens was attributed to the secretion of estrogens by the ovaries as well as their autocrine and paracrine action, and an increased aromatase activity in EM lesions that supports local E_2_ synthesis (15, 17, 39–41). Additional changes in estrogen synthesis, as well as their metabolic and receptor pathways, have also been reported in support of an enhanced local production and action in EM lesions, creating a hyperestrogenic environment that affects hormone receptor function (38, 42–49). These changes may be reflected in circulation of EM cases with higher levels of E_2_ and/or its metabolites. In our study, 2OH-3MeO-E_1_ was associated with an increased risk of EM. This observation is consistent with elevated COMT expression in EM lesions (50) leading to the formation of 2OH-3MeO-E_1_ from its precursor 2OH-E_1_ (23), potentially contributing to higher systemic levels of this metabolite. In support, we showed that endometrial tissue can contribute to systemic estrogen levels in the context of endometrial cancer that significantly declined after surgery (29). In addition to significant higher circulating levels of 2OH-3MeO-E_1_metabolites in EM cases compared to controls, higher levels of 2MeO-E_1_ and the sum of MeOs were observed but they did not reached significance. The biological properties of 2OH-3MeO-E_1_ have been poorly studied. We further noted that this metabolite was less correlated in circulation with the other estrogen derivatives and particularly in control subjects, suggesting a dysregulation in the presence of EM lesions associated with its precursors such as 2OH-E_1_, with a higher correlation coefficient for this metabolite in EM cases at 0.20 (*P*<0.05). In fact, 2OH-3MeO-E_1_ was higher in cases compared to controls, supporting a potential EM origin. Consistent with our observation, a previous study evaluated a subset of estrogen metabolites in preoperative urine samples of 62 EM cases and 52 controls and found increased levels of the 2OH-3MeO-E_1_ precursor 2OH-E_1_ (51). Our findings that the 2OH pathway is significantly more elevated in ovarian EM cases is also consistent with a study that used proton nuclear magnetic resonance (H-NMR) spectroscopy to investigate potential non-invasive metabolomic markers in 31 infertile women with stage II and III EM cases and 15 healthy or control women (52). They found that levels of the antiangiogenic 2MeO-E_1_/E_2_ metabolites were higher in EM cases compared to controls. The enrichment of the 2OH metabolic pathway in ovarian EM cases is supported by higher tissular levels of CYP1A1, involved in the conversion of E_1_ and E_2_ to 2OH-E_1_ and 2OH-E_2,_ reported to be 4-fold higher in the ovarian EM group (50, 53). Inversely, the 16OH pathway was inversely associated with EM, consistent with downregulation of the involved enzyme pathways (CYP3A) by inflammation (54, 55).

An estrogenic environment may be associated with more severe pain symptoms (13). Hence, the association between estrogen levels and pain outcomes may not be related only to the effect of estrogens, as E_2_ metabolites have been documented to present receptor-independent biological activities and may contribute to the maintenance of the inflammatory milieu (16). Previous reports revealed that elevated ERβ is associated with proliferation, inflammation and pain transmission (46, 56, 57), coherent with the positive correlation observed in this study between 16keto-E_2_ and dyspareunia in EM cases. However, the negative association between 16αOH-E_1_ and pelvic, abdominal and back pain suggests more complex relationships. A component of pain in EM was shown to be related to inflammatory damage of nerve fibers with neuroprotective roles for ERβ (58–61). Also, a dysregulation of both the ERα and ERβ expression pathways was observed in the ectopic endometrium in EM compared to normal endometrium in favor of a superior ERβ to ERα ratio (46, 62, 63). 16-hydroxylated derivatives are amongst E_2_ metabolites known to bind the ERβ receptor (24), which may explain the observed association with pain. Other studies showed that the ERα was correlated with symptoms in deep infiltrating EM (64) and that it could favor hyperalgesia by altering calcium release (61). Since the 2OH metabolites are known to bind ERα (24), this could explain the association between pain outcomes and 2OH metabolites, such as 2OH-3MeO-E_1_. Additional studies are required to uncover the precise biological function of the 2OH-3MeO-E_1_ metabolite.

This pilot study provides a comprehensive quantification of estrogens in the circulation of EM cases and controls based on a sensitive mass-spectrometry assay. It is comprised of a significant sample size, surgical and histologic confirmation of case and control status, adjustment for confounding factors and examination of pain symptoms. A limitation is the fact that the control group also included patients with gynecological conditions other than EM, which may influence the hormonal milieu (40, 65). Although cases and controls differed in confounding factors such as menstrual phase, these variables were included in the multivariate model for EM risk. Exploratory analyses in relation to pain symptoms were adjusted for age and BMI. Additional studies could provide levels of progesterone and its metabolites, shown to be dysregulated in EM and recognized to counteract the effect of E_2_ (48, 66), whereas the endometriotic intratissue estrogen levels may not reflect the corresponding systemic levels. Due to the exploratory nature of the study, no correction for multiple testing was applied, but our initial findings warrant replication in other cohorts.

## Conclusions

We conclude that the 2OH-3MeO-E_1_ metabolite represents a potential adverse feature of EM and that the 2OH pathway is associated with the risk of ovarian endometriotic lesions. Data also suggest an association between the 2OH metabolic pathway and the risk of unfavorable pain outcomes.

## Data availability statement

The original contributions presented in the study are included in the article/**Supplementary Material**. Further inquiries can be directed to the corresponding authors.

## Ethics statement

The studies involving human participants were reviewed and approved by National Medical Ethics Committee in Slovenia (#0120-127/2016/6) and the Ethics committee of the CHUQc – Université Laval (#2012-993). The patients/participants provided their written informed consent to participate in this study.

## Author contributions

Study concept and design: TR, CG. Patient recruitment and clinical data: MP, AV, JO, TR. Conducted experiments and mass spectrometry: PC, VT. Statistical analyses: DS. Drafting of the manuscript: J-PE, CG. Critical revision of the manuscript for important intellectual content: All authors. Obtaining funding: TR, CG.
