# Supplementary material for: Circulating estradiol and its biologically active metabolites in endometriosis and in relation to pain symptoms
Source: Front Endocrinol (Lausanne). 2023 Jan 18;13:1034614. doi: 10.3389/fendo.2022.1034614 (PMC9891204; doi:10.3389/fendo.2022.1034614)
Supplement: Supplementary file 1 [file DataSheet_1.pdf]

Supplementary Table 1. Patient's characteristics related to pain symptoms.

Pain symptoms	Cases (n=209)		Controls (n=115) Benign pathologies (n=79)		Controls (n=115) Healthy controls (n=36)	
	**n**	**%**	**n**	**%**	**n**	**%**
**Pelvic, abdominal or back pain**						
Yes	92	44.0	22	27.8	6	16.7
No	115	55.0	55	69.6	29	80.6
NA	2	1.0	2	2.5	1	2.8
**Menstrual pain frequency**						
1- never	4	1.9	3	3.8	16	44.4
2 - almost never	12	5.7	13	16.5	3	8.3
3 - sometimes	51	24.4	14	17.7	14	38.9
4 - quite often	33	15.8	16	20.3	0	0.0
5 - very often	108	51.7	31	39.2	2	5.6
NA	1	0.5	2	2.5	1	2.8
**Menstrual pain intensity**						
1- No	16	7.7	12	15.2	13	36.1
2- Slight (small impact on the ability to work)	49	23.4	33	41.8	5	13.9
3- Medium (1 day in bed, partially unfit for work)	63	30.1	14	17.7	7	19.4
4- Strong (more than 1 day in bed, unable to work)	68	32.5	16	20.3	1	2.8
NA	13	6.2	4	5.1	10	27.8
**Menstrual pain score (0-10)**						
0-2	11	5.3	13	16.5	NA	NA
3-5	37	17.7	18	22.8	NA	NA
6-8	57	27.3	28	35.4	NA	NA
9-10	23	11.0	6	7.6	NA	NA
NA	81	38.8	14	17.7	NA	NA
**Pain during sexual intercourse frequency**						
1- never	52	24.9	29	36.7	21	58.3
2 - almost never	42	20.1	17	21.5	5	13.9
3 - sometimes	57	27.3	24	30.4	5	13.9
4 - quite often	27	12.9	4	5.1	0	0.0
5 - very often	28	13.4	3	3.8	0	0.0
NA	3	1.4	2	2.5	5	13.9
**Pain during sexual intercourse intensity**						
0- No	52	24.9	38	48.1	NA	NA
1- Slight	43	20.6	17	21.5	NA	NA
2- Medium (each sexual intercourse)	27	12.9	8	10.1	NA	NA
3- Strong (without sexual intercourses due to pain)	2	1.0	1	1.3	NA	NA
NA	85	40.7	15	19.0	NA	NA
**Pain during sexual intercourse - score (0-10)**						
0-2	58	27.8	38	48.1	NA	NA
3-5	42	20.1	18	22.8	NA	NA
6-8	21	10.0	6	7.6	NA	NA
9-10	2	1.0	0	0.0	NA	NA
NA	86	41.1	17	21.5	NA	NA
**Pain during urination/defecation**						
1- never	115	55.0	55	69.6	31	86.1
2 - almost never	29	13.9	14	17.7	1	2.8
3 - sometimes	45	21.5	5	6.3	3	8.3
4 - quite often	17	8.1	3	3.8	0	0.0
5 - very often	2	1.0	0	0.0	0	0.0
NA	1	0.5	2	2.5	1	2.8

Supplementary Table 2. Percentage (%) of detection of circulating levels of 16 estrogen derivatives quantified by mass spectrometry in the study cohort (n=341 women).

Steroids	Detection (%)
Estrogens	
**E_1**	**100.0**
**E_2**	**96.8**
2OH estrogens	
**2OH-E_1**	**95.6**
**2OH-E_2**	**70.7**
4OH estrogens	
**4OH-E_1**	**66.0**
4OH-E_2	10.9
16OH estrogens	
**16αOH-E_1**	**95.6**
**16epi-E_3**	**74.5**
**16keto-E_2**	**94.7**
17epi-E_3	10.0
**E_3**	**98.2**
MeO estrogens	
**2MeO-E_1**	**89.1**
2MeO-E_2	12.0
**2OH-3MeO-E_1**	**47.2**
4MeO-E_1	8.5
4MeO-E_2	2.3

The lower limit of quantification (LLOQ) was 5 pg/mL. Steroids retained for subsequent individual analyses are indicated in bold (11 out of the 16 measured).

Supplementary Table 3. Steroid levels according to surgical stages of disease in 209 endometriosis cases.

Steroids (pg/mL)	Median (10-90%)			
	Stage 1 n=49	Stage 2 n=38	Stage 3 n=67	Stage 4 n=40
E_1	862.00 (188.00-3260.00)	1099.50 (172.00-4080.00)	848.00 (285.00-2130.00)	784.00 (175.50-2085.00)
E_2	142.00 (9.44-449.00)	181.00 (8.04-484.00)	138.00 (19.80-393.00)	127.00 (6.95-271.50)
2OH-E_1	115.00 (6.10-268.00)	113.00 (7.76-359.00)	80.90 (16.80-209.00)	95.56 (14.97-202.50)
2OH-E_2	15.70 (2.50-39.70)	11.45 (2.50-50.90)	7.92 (2.50-39.20)	9.33 (2.50-34.50)
4OH-E_1	10.00 (2.50-24.60)	9.24 (2.50-35.10)	8.59 (2.50-24.60)	8.16 (2.50-23.75)
16αOH-E_1	34.80 (5.21-154.00)	53.90 (6.42-251.00)	29.20 (8.83-153.00)	40.55 (6.44-128.50)
16epi-E_3	8.41 (2.50-39.50)	8.12 (2.50-25.60)	8.71 (2.50-30.90)	7.70 (2.50-23.15)
16keto-E_2	26.60 (7.85-85.20)	46.50 (5.68-137.00)	31.90 (10.00-163.00)	29.60 (7.84-102.85)
E_3	69.60 (9.06-389.00)	90.90 (13.40-233.00)	70.50 (20.70-240.00)	61.00 (16.05-287.00)
2MeO-E_1	22.70 (2.50-73.30)	31.05 (2.50-91.50)	19.30 (2.50-88.80)	23.80 (6.23-81.35)
2OH-3MeO-E_1	2.50 (2.50-16.00)	5.14 (2.50-17.40)	2.50 (2.50-14.30)	6.24 (2.50-15.20)
**Sums**				
Parental (P)	1039.00 (196.86-3744.00)	1280.00 (180.04-4470.00)	1015.00 (345.60-2276.00)	866.75 (379.08-2381.00)
2OH	139.70 (8.60-296.80)	123.70 (10.26-438.70)	92.00 (19.30-251.60)	104.20 (17.47-240.80)
4OH	12.50 (5.00-32.30)	12.86 (5.00-41.70)	11.09 (5.00-32.06)	10.66 (5.00-29.11)
16OH	147.41 (32.61-632.70)	220.61 (27.95-681.30)	152.71 (45.00-656.90)	143.88 (33.78-498.10)
MeOs	41.62 (12.50-95.00)	47.20 (18.74-116.70)	33.25 (15.06-110.11)	40.25 (24.23-100.60)
OHs	283.17 (59.50-973.20)	366.37 (49.93-850.16)	290.03 (80.69-933.72)	307.40 (69.51-687.46)
All CEs	348.97 (79.18-1056.64)	408.54 (62.43-1052.56)	326.61 (105.77-1038.35)	342.42 (100.48-773.28)
**Ratios**				
2OH/P	0.09 (0.03-0.23)	0.10 (0.03-0.22)	0.09 (0.04-0.18)	0.10 (0.03-0.26)
4OH/P	0.01 (0.00-0.03)	0.01 (0.00-0.04)	0.01 (0.00-0.03)	0.01 (0.01-0.03)
16OH/P	0.14	0.16	0.15	0.15

	(0.06-0.50)	(0.09-0.36)	(0.06-0.38)	(0.06-0.56)
CEs/P	0.31 (0.18-0.70)	0.31 (0.18-0.75)	0.36 (0.17-0.62)	0.36 (0.16-0.81)
2OH/4OH	8.53 (1.00-27.94)	9.27 (1.91-26.80)	7.60 (3.01-17.58)	8.78 (2.48-21.27)
2OH/16OH	0.65 (0.12-1.98)	0.56 (0.19-1.43)	0.67 (0.13-1.44)	0.80 (0.15-2.28)
2OH/MeOs	2.86 (0.40-4.52)	2.49 (0.80-4.46)	2.38 (0.98-3.93)	2.35 (0.88-4.15)
4OH/16OH	0.08 (0.02-0.25)	0.07 (0.03-0.21)	0.08 (0.01-0.28)	0.10 (0.01-0.34)
4OH/MeOs	0.29 (0.16-0.62)	0.24 (0.11-0.70)	0.33 (0.14-0.63)	0.27 (0.12-0.52)
OHs/MeOs	6.77 (2.26-15.43)	6.49 (2.51-15.19)	6.60 (2.83-17.46)	5.50 (2.90-20.04)
2OH-E_1/16αOH-E_1	2.50 (0.31-10.84)	1.84 (0.38-6.48)	2.97 (0.40-7.97)	2.61 (0.41-9.69)

The sum of parental estrogens (P) includes E_1 and E_2. The sum of hydroxy derivatives (OHs) includes 2OH, 4OH and 16OH. The sum of all catechol estrogens (CEs) includes OHs and MeOs. Data represents bivariate analysis corrected with Tukey and adjusted for age and BMI. *P*-values were obtained using log transformed data with the F test. No evidence of significant associations was observed. The rASRM classification was used to determine the surgical stage.

Supplementary Table 4. Significant associations between steroids and pain symptoms in EM cases (n=209).

Steroids	Comparator Medians in pg/mL (n)	Outcome Medians in pg/mL (n)	OR (95%CI)	*P*-value
**Abdominal, pelvic, and back pain (n=207)**				
**16αOH-E$_1$**	No 42.60 (115)	Yes 26.15 (92)	0.55 (0.31-0.97)	**0.038**
**2OH-3MeO-E$_1$**	No 2.50 (115)	Yes 5.99 (92)	1.86 (1.06-3.27)	**0.032**
**Dysmenorrhea (frequency) (n=208)**				
**Sum P**	Infrequent 857.00 (67)	Frequent 1064.90 (141)	1.90 (1.03-3.51)	**0.041**
**E$_1$**	Infrequent 680.00 (67)	Frequent 918.00 (141)	1.80 (0.97-3.32)	0.061
**CEs/P**	Infrequent 0.41 (67)	Frequent 0.31 (141)	0.55 (0.30-1.01)	0.053
**16OH/P**	Infrequent 0.17 (67)	Frequent 0.14 (141)	0.58 (0.32-1.07)	0.080
**Dysmenorrhea (intensity) (n=74)**				
**Ratio 4OH/P**	Mild 0.01 (45)	Moderate to severe 0.01 (29)	1.95 (1.05-3.61)	**0.035**
**Sum MeO**	Mild 37.90 (45)	Moderate to severe 41.78 (29)	1.88 (1.00-3.45)	0.052
**Dyspareunia (intensity) (n=124)**				
**16keto-E$_2$**	Mild 26.90 (95)	Moderate to severe 46.60 (29)	2.40 (1.02-5.62)	**0.045**
**Score of dyspareunia (n=128)**				
**CEs/P**	Mild 0.35 (48)	Moderate to severe 0.29 (80)	0.49 (0.23-1.04)	0.064
**Dysuria or dyschezia (frequency) (n=208)**				
**2MeO-E$_1$**	Infrequent 24.60 (189)	Frequent 11.80 (19)	0.42 (0.16-1.12)	0.083
**4OH-E$_1$**	Infrequent 8.87 (189)	Frequent 2.50 (19)	0.32 (0.12-0.89)	**0.028**
**2OH-E$_2$**	Infrequent 11.00 (189)	Frequent 2.50 (19)	0.34 (0.11-1.08)	0.068
**Sum 4OH**	Infrequent 11.42 (189)	Frequent 5.00 (19)	0.42 (0.16-1.12)	0.083

The sum of parental estrogens (P) includes E_1 and E_2. The sum of hydroxy derivatives (OHs) includes 2OH, 4OH and 16OH. The sum of all catechol estrogens (CEs) includes OHs and MeOs. Odds ratios (OR) and their *P*-values were obtained using a logistic regression model adjusted for

age and BMI. *P*-values <0.05 are in bold and findings with *P* <0.10 are also displayed. There were no significant associations or trends in controls for abdominal, pelvic, and back pain. The number of cases with data on clinical outcomes is identified next to each outcome. There were no significant association for the "score of dysmenorrhea" and the "dyspareunia (frequency)" outcomes.
